# Mycotic Pseudoaneurysm Formation at the Cannulation Site in the Ascending Aorta

**DOI:** 10.7759/cureus.19283

**Published:** 2021-11-05

**Authors:** Arminder Singh, William Sanchez-Garcia, Robert Maughan, Divyang R Patel, Amol Bahekar

**Affiliations:** 1 Internal Medicine, Cape Fear Valley Medical Center, Fayetteville, USA; 2 Cardiology, Cape Fear Valley Medical Center, Fayetteville, USA; 3 Cardiothoracic Surgery, Cape Fear Valley Medical Center, Fayetteville, USA

**Keywords:** dyslipidemia, coronary artery bypass grafting (cabg), aorta, hypertension, chest pain

## Abstract

The formation of mycotic pseudoaneurysms in the ascending aorta is a rare but sometimes fatal complication after open-heart surgery, requiring cardiopulmonary bypass (CPB). There has been little cited about this rare complication. We present a case of a 51-year-old man who developed a mycotic pseudoaneurysm in the ascending aorta at a previous aortic cannulation site nine years after coronary artery bypass surgery. The patient presented to the emergency department with two weeks of worsening substernal chest pain and was found to have pseudoaneurysm in the anterior wall of the ascending aorta on chest computed tomography angiography (CTA) during his chest pain workup. The patient's blood cultures grew methicillin-susceptible *Staphylococcus aureus* (MSSA). During the hospital course, the patient's respiratory status worsened, and repeat CTA revealed enlargement of the pseudoaneurysm arising from the anterior proximal arch of the aorta. Chest X-ray obtained because of hypoxia demonstrated widening of the upper mediastinum, which appeared increased compared with the previous exam. Because of concern for rupture of an aneurysm, the patient was taken to the operating room for redo sternotomy and repair of the pseudoaneurysm with femoral artery cannulation for cardiopulmonary bypass. The patient completed eight weeks of IV nafcillin, and rifampin was added to decrease biofilm formation.

## Introduction

Aneurysms are broken down into two categories of true or false (pseudoaneurysm) [1]. Pseudoaneurysms are commonly caused by trauma, inflammation, and iatrogenic factors, such as drainage, surgery, and percutaneous biopsy [1,2]. True aneurysms contain all three layers of the aortic wall (intima, media, and adventitia), but in pseudoaneurysms, there are fewer than three layers [1]. Pseudoaneurysms arise in the arterial wall continuity that allows blood to dissect the tissue, creating a perfused sac around the damaged artery with persistent communication with the arterial lumen [2]. The common risk factors for pseudoaneurysm include atherosclerosis, increasing age, diabetes mellitus, malignancy, HIV infection, and immunosuppressive conditions [1]. A mycotic pseudoaneurysm can develop when a pre-existing aneurysm becomes infected, and bacterial infections are implicated in approximately 13% of mycotic aneurysms [1-3]. During inflammation, there is a hematogenous spread of the microorganism into the vasa vasorum of the vessel wall resulting in inflammation that destroys the adventitia and muscularis, leading to a rupture of the artery into the surrounding tissue with contained rupture and formation of mycotic pseudoaneurysm [2]. Here, we describe a case of mycotic pseudoaneurysm in the ascending aorta at a previous aortic cannulation site with methicillin-susceptible *Staphylococcus aureus* (MSSA) bacteremia.

## Case presentation

A 51-year-old gentleman presented to the emergency department (ED) with a two-week history of worsening substernal chest pain. He had a history of coronary artery disease status post-coronary artery bypass surgery nine years before presentation and percutaneous coronary interventions (PCI) with stenting six months before presentation. Additionally, his past medical history also included hypertension, hyperlipidemia, being a 30-pack-per-year tobacco smoker, chronic pain disorder, and anxiety disorder. The patient presented with chest pain described as sharp, non-radiating, aggravated by being in a supine position, and alleviated by analgesics. He had also complained of subjective fevers and generalized weakness. Physical examination demonstrated an anxious male patient with normal vital signs and no chest wall tenderness upon palpation of the thorax.

**Figure 1:**
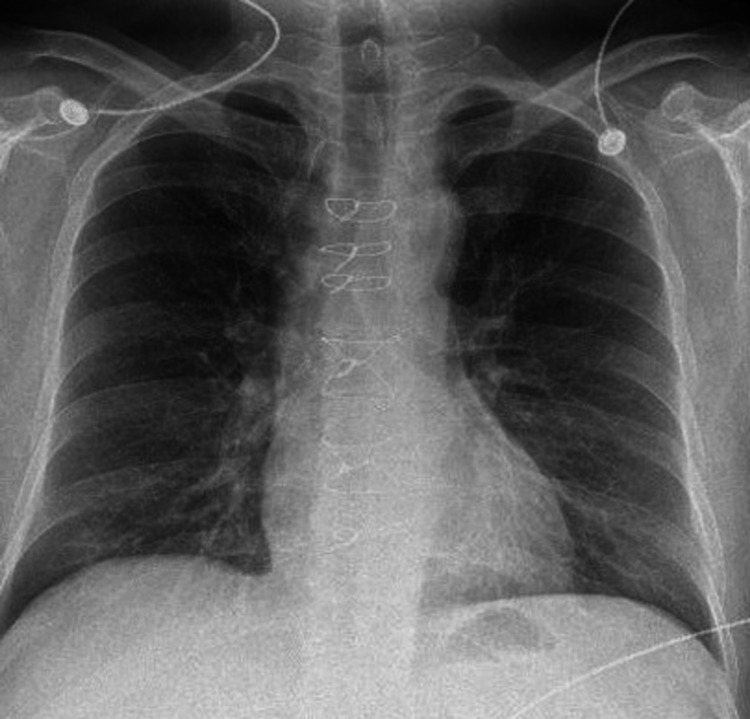
Posteroanterior (PA) chest radiography demonstrating an increasing nodular infiltrate at the left lung apex and post-operative changes in addition to chronic fibrotic.

**Figure 2:**
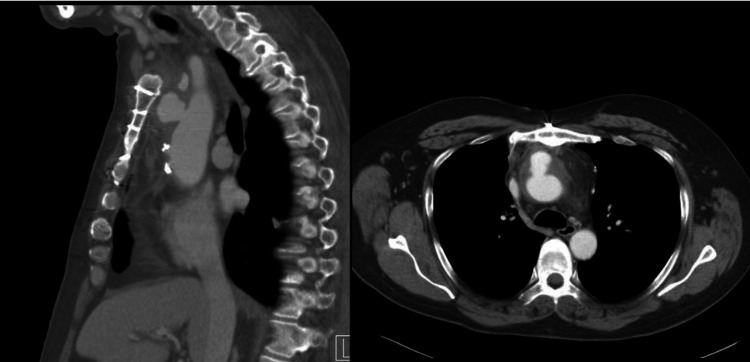
Chest computed tomography with contrast demonstrating a pseudoaneurysm in the anterior wall of the ascending aorta measuring 2.1 x 1.9 cm. The neck of the aneurysm measures 1.0 cm.

Laboratory test results were significant for the followinmediastinal g: leukocytosis of 17.9 x 10^3/uL, a hemoglobin level of 11.7 g/dL, C-reactive protein (CRP) measured at 181.0 mg/L, and undetectable cardiac troponin (TnI) three hours apart. Serum electrolytes, blood urea nitrogen, and creatinine were within normal limits. A 12-lead electrocardiogram demonstrated sinus rhythm with probable left atrial enlargement and prolonged QT interval. On the day of admission, a posteroanterior (PA) chest radiograph demonstrated an increasing nodular infiltrate at the left lung apex and postoperative changes in addition to chronic fibrotic changes (Figure 1). On the day of admission, chest computed tomography angiography (CTA) demonstrated a pseudoaneurysm in the anterior wall of the ascending aorta measuring 2.1 x 1.9 cm, whose neck measured 1.0 cm (Figure 2). Subsequent two-dimensional transthoracic echocardiography (TTE) demonstrated normal biventricular systolic function with a left ventricular (LV) ejection fraction greater than 55%, mild diastolic dysfunction with normal estimated left atrial pressure, and no significant valvular abnormalities. Transesophageal echocardiography (TEE) demonstrated normal aorta in visualized portions and no evidence of valve vegetation or septal defects. Subsequently, methicillin-susceptible *Staphylococcus aureus* (MSSA) was isolated in blood cultures. The patient has no previous history of being bacteremic.

**Figure 3:**
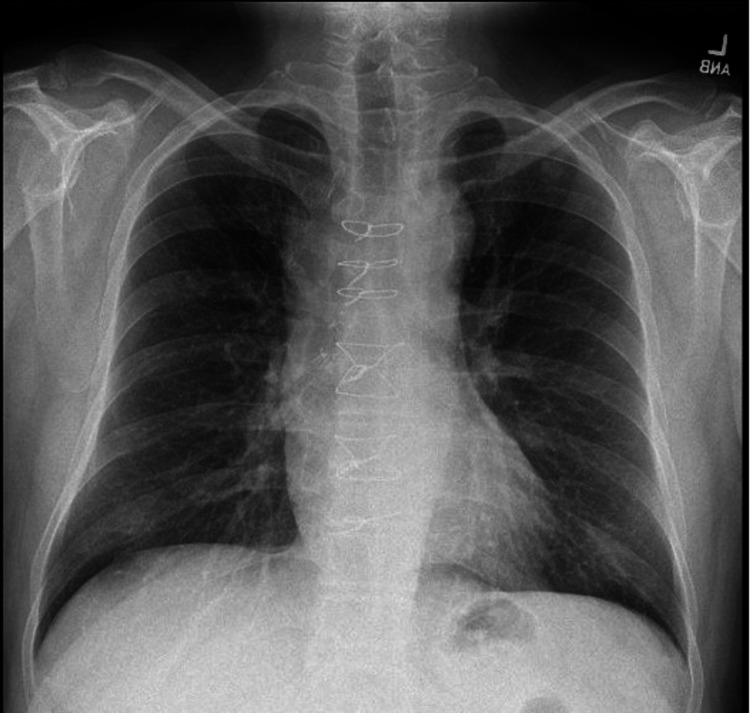
Repeat posteroanterior chest radiograph demonstrating wider upper mediastinum.

**Figure 4:**
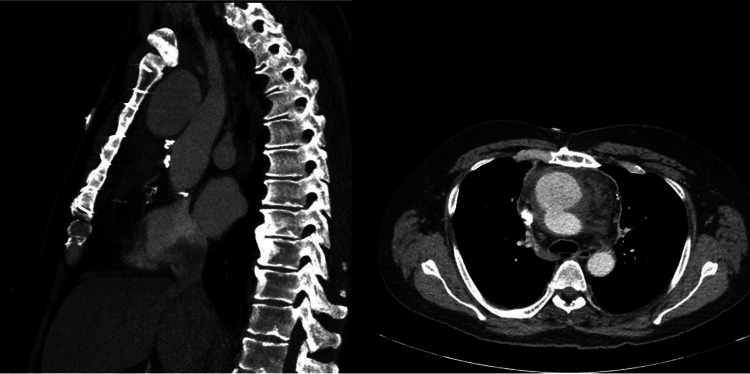
The follow-up computed tomography angiography with and without contrast demonstrating significant enlargement of the pseudoaneurysm arising from the anterior margin.

Cardiothoracic surgery was consulted and recommended awaiting workup results that included TTE, TEE, blood cultures, and cardiology consult. Cardiology was consulted and suggested no plan for left heart catheterization given TTE with normal ejection fraction (EF) and no wall motion abnormalities. The cardiothoracic surgery department recommended source control and identification of bacteremia before surgical intervention. However, while awaiting source control and identification of bacteremia on day four of admission, the patient became hypoxic. Due to the patient's worsening hypoxia and increased oxygen requirement, a repeat chest radiography demonstrated widening of the upper mediastinum, which appeared increased compared to the previous exam (Figure 3). Subsequently, after the chest radiography, computed tomography angiography with and without contrast demonstrated significant enlargement of the pseudoaneurysm arising from the anterior proximal arch of the aorta, measuring up to 4.8 x 4.1 x 5.9 cm (Figure 4).

**Figure 5:**
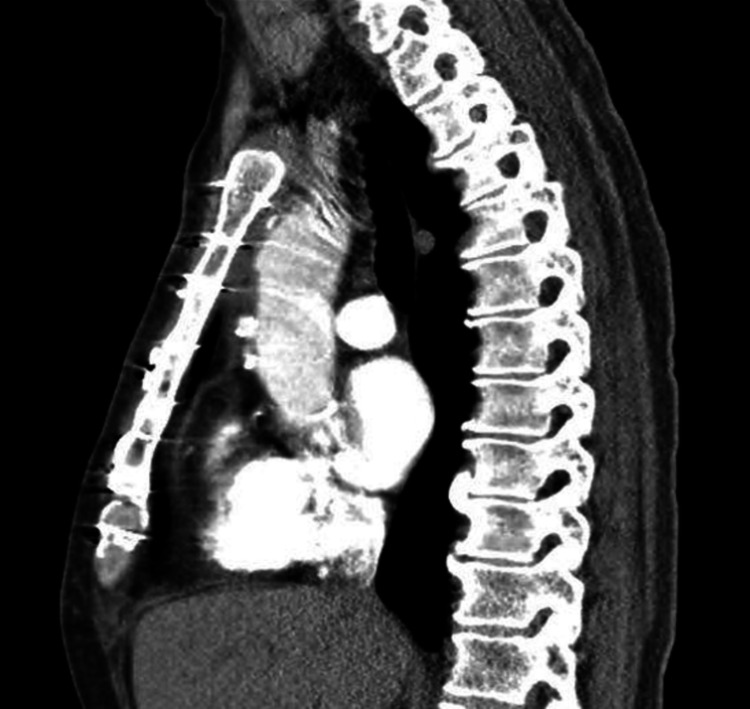
Repeat chest tomography revealed no aneurysm or effusions.

The patient was urgently taken to the operating room for redo sternotomy and repair of the pseudoaneurysm with femoral artery cannulation for cardiopulmonary bypass. A large defect was present in the ascending aorta at the site of the previous cannulation. Significant inflammation and infection were present within the mediastinum. The perforation area was sharply debrided then closed under circulatory arrest using a hemashield patch and a suture of 5-0 Prolene (Ethicon, Raritan, New Jersey). The circulation was restored, and active rewarming was performed as he was weaned off the cardiopulmonary bypass. During the operation, the patient had significant coagulopathy and required multiple plasma, platelet, and cryoprecipitate transfusions. Two mediastinal chest tubes were placed and gradually removed postoperatively at the end of the first week. The total hypothermic circulatory arrest time was 39 minutes, with a total bypass time of 228 minutes. Concomitantly, the patient was started on nafcillin for MSSA bacteremia. Repeat chest tomography four days after surgery revealed no aneurysm or effusions (Figure 5). The patient was treated with a total of eight weeks of IV nafcillin, and rifampin was added to decrease biofilm formation. The patient was discharged on postoperative day 20 and had uncomplicated follow-ups.

## Discussion

Development of mycotic aneurysms is a rare complication of open-heart surgery requiring cannulation for cardiopulmonary bypass [1-3]. It is even rarer for the aortic cannulation site to be infected. The most commonly isolated pathogen is *Staphylococcus aureus* [1-4]. Other pathogens implicated with ascending aortic aneurysm infection are *Staphylococcus epidermidis* and *Propionibacterium acnes* [4]. The most common locations of pseudoaneurysm origination are the proximal anastomosis, suture lines, and cannulation sites [1,2,4]. The intraoperative transesophageal echocardiography characteristics of pseudoaneurysms include a thin-walled cavity, expansion during systole, and collapse during diastole, and TEE can be used to confirm the diagnosis of pseudoaneurysm once suggested by magnetic resonance imaging or computed tomography [1,4]. However, in this case, the pseudoaneurysm's location was very distal in the ascending aorta, making visualization by TEE very difficult.

Pseudoaneurysms are most often caused by traumatic injuries. However, they can also be caused by inflammation and iatrogenic factors, such as drainage, surgery, and percutaneous biopsies [3,5]. A mycotic pseudoaneurysm can develop when a pre-existing aneurysm becomes infected, and bacterial infections are implicated in approximately 13% of mycotic aneurysms [1,3]. In this case, pseudoaneurysm was infected by methicillin-sensitive *Staphylococcus aureus*. Risk factors for pseudoaneurysm formation include atherosclerosis, advanced age, diabetes mellitus, malignancy, HIV infection, and immunosuppressive conditions [3,4,6]. In this case, the patient had an extensive atherosclerotic disease.

Repeat sternotomy carries a high risk of fatal hemorrhage due to the aneurysm's tendency to adhere to the sternum [5]. In this case, the cardiopulmonary bypass was commenced before the sternotomy by femoral artery cannulation to prevent such complications [5]. Endovascular repair of the defect is commonly preferred over open repair when viable; however, in this case, due to the rapid clinical deterioration and the pseudoaneurysm's location, the endovascular graft-stent repair option was deemed not feasible. Along with the open or percutaneous repair, the therapeutic mainstay is prolonged culture-specific intravenous antibiotics for four to six weeks [1].

## Conclusions

Mycotic pseudoaneurysms are a very rare complication following open-heart surgery, carrying high mortality. Limited information exists on this complication, specifically on infection of a pseudoaneurysm. It is vital to keep aortic aneurysms as part of differential diagnosis when investigating chest pain in a patient with a history of heart surgery. Further surveillance and research are needed to assess the incidence of mycotic pseudoaneurysms in the general population with chest pain or recurrent chest pain with a history of previous heart surgery. This case highlights the importance of keeping pseudoaneurysm formation in the list of diagnostic considerations for patients with a history of open-heart surgery presenting with chest pain.
